# Chaperone-Mediated Autophagy Protein BAG3 Negatively Regulates Ebola and Marburg VP40-Mediated Egress

**DOI:** 10.1371/journal.ppat.1006132

**Published:** 2017-01-11

**Authors:** Jingjing Liang, Cari A. Sagum, Mark T. Bedford, Sachdev S. Sidhu, Marius Sudol, Ziying Han, Ronald N. Harty

**Affiliations:** 1 Department of Pathobiology, School Veterinary Medicine, University of Pennsylvania, Philadelphia, PA, United States of America; 2 Laboratory of Animal Infectious Diseases, College of Animal Sciences and Veterinary Medicine; State Key Laboratory for Conservation and Utilization of Subtropical Agro-Bioresources, Guangxi University, Nanning, Guangxi, China; 3 Department of Epigenetics & Molecular Carcinogenesis, M.D. Anderson Cancer Center, University of Texas Smithville, Smithville, TX, United States of America; 4 Department of Molecular Genetics, University of Toronto, Toronto, ON, Canada; 5 Department of Physiology, National University of Singapore, Mechanobiology Institute and Institute for Molecular and Cell Biology (IMCB, A*STAR), Republic of Singapore; Georgia State University, UNITED STATES

## Abstract

Ebola (EBOV) and Marburg (MARV) viruses are members of the *Filoviridae* family which cause outbreaks of hemorrhagic fever. The filovirus VP40 matrix protein is essential for virus assembly and budding, and its PPxY L-domain motif interacts with WW-domains of specific host proteins, such as Nedd4 and ITCH, to facilitate the late stage of virus-cell separation. To identify additional WW-domain-bearing host proteins that interact with VP40, we used an EBOV PPxY-containing peptide to screen an array of 115 mammalian WW-domain-bearing proteins. Using this unbiased approach, we identified BCL2 Associated Athanogene 3 (BAG3), a member of the BAG family of molecular chaperone proteins, as a specific VP40 PPxY interactor. Here, we demonstrate that the WW-domain of BAG3 interacts with the PPxY motif of both EBOV and MARV VP40 and, unexpectedly, inhibits budding of both eVP40 and mVP40 virus-like particles (VLPs), as well as infectious VSV-EBOV recombinants. BAG3 is a stress induced protein that regulates cellular protein homeostasis and cell survival through chaperone-mediated autophagy (CMA). Interestingly, our results show that BAG3 alters the intracellular localization of VP40 by sequestering VP40 away from the plasma membrane. As BAG3 is the first WW-domain interactor identified that negatively regulates budding of VP40 VLPs and infectious virus, we propose that the chaperone-mediated autophagy function of BAG3 represents a specific host defense strategy to counteract the function of VP40 in promoting efficient egress and spread of virus particles.

## Author Summary

The unprecedented magnitude and scope of the catastrophic 2014–2015 EBOV outbreak in West Africa, and its continued global emergence underscores the urgent need to better understand the biology and pathogenesis of this zoonotic pathogen. We have identified BAG3 as a novel and functional host VP40 interactor that negatively regulates VP40 VLP and virus egress in a PPxY/WW-domain dependent manner. As a cell survival protein and key regulator of chaperone-mediated autophagy (CMA), BAG3 sequesters EBOV and MARV VP40 away from the site of budding at the plasma membrane, and thus may represent a novel host defense strategy to combat filovirus VP40-mediated egress and spread.

## Introduction

Ebola (EBOV) and Marburg (MARV) viruses are virulent pathogens that cause severe hemorrhagic disease in humans and non-human primates. There are currently no FDA approved vaccines or antiviral drugs to prevent or treat infections by these Category A NIAID priority pathogens [1]. The recent catastrophic outbreak of EBOV in West Africa underscores the urgent need to better understand the biology and pathogenesis of this global public health threat, and to decipher the molecular mechanisms by which EBOV interacts with the host to cause disease.

The filovirus matrix protein VP40 is the most abundant protein in the virion and is essential for virus assembly and egress. Indeed, expression of VP40 alone is sufficient to form virus-like particles (VLPs), which are morphologically indistinguishable from infectious virions and are released from mammalian cells in a manner that recapitulates the release of authentic virus [2–6]. Although not required for EBOV replication [7], Late (L) domains (which contain PTAP and/or PPxY amino acid sequence motifs) are conserved within EBOV and MARV VP40 and promote efficient egress of VLPs and virus by recruiting host proteins that facilitate virus-cell separation [3,4,6,8–11]. For example, EBOV and MARV VP40 L-domains hijack specific host proteins associated with the ESCRT pathway, including Tsg101, Alix, and Nedd4 [3,6,8–13].

Viral proteins bearing PPxY motif each interact with a unique repertoire of WW-domain bearing host proteins with diverse functions [14–22]. For example, the PPxY L-domain within eVP40, mVP40, and other viral matrix proteins interacts specifically with WW-domains of: 1) host Nedd4; a HECT family E3 ubiquitin ligase that is linked with the cellular ESCRT machinery, 2) host ITCH; a HECT family E3 ubiquitin ligase involved in immune regulation and inflammatory signaling, and 3) host IQGAP1; a multifunctional scaffolding protein involved in regulating cell motility, actin polymerization, and filopodia formation [2,23–38]. In general, these previously characterized viral PPxY/WW-domain interactions promote efficient virus production.

Here, we sought to identify additional WW-domain bearing proteins that interact with the eVP40 PPxY motif by screening a GST array of 115 host proteins containing one or more WW-domains [39] with an EBOV PPxY-containing peptide. Using this unbiased approach, we identified WW-domain containing protein BAG3 as a novel eVP40 interactor. BAG3 is a stress-induced molecular co-chaperone involved in regulating cellular protein homeostasis by CMA. Since in general, viral PPxY-containing proteins tend to bind WW-domains with good specificity and selectivity [40], our identification of BAG3 suggests that this protein may play a biologically relevant role in the lifecycle of EBOV. Indeed, we confirmed that hypothesis by first using co-IP to validate the specificity of the PPxY/WW-domain physical interaction between VP40 (both eVP40 and mVP40) and BAG3, and functionally demonstrated that expression of BAG3 inhibited VP40 VLP production, as well as budding of a VSV recombinant virus containing the EBOV VP40 PPxY L-domain motif. To our knowledge, this is the first identification of a VP40-interacting mammalian WW-domain bearing protein that negatively regulates budding. Mechanistically, our data suggest that BAG3 binds VP40 and not only sequesters it away from the site of budding at the plasma membrane, but also directs a fraction of VP40 into aggresomes, thus reducing VLP egress.

## Results

### Screening of proline-rich motif reading array identifies BAG3 as an EBOV VP40 PPxY interactor

**Fig 1:**
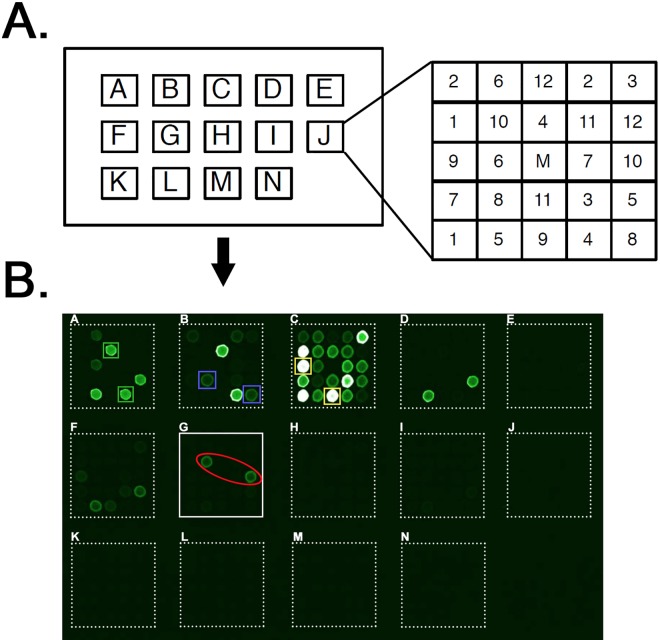
Proline-rich reading array identifies BAG3 as an eVP40 interactor. **A)** Schematic diagram of the “proline-rich” reading array chip. Each lettered square contains 12 numbered WW- and/or SH3 GST fusion domains in duplicate. A mock (M) GST sample is in the center of each square. **B)** The fluorescent pattern following binding of the EBOV VP40-WT biotinylated peptide to the array. The fluorescent spots indicate a positive peptide/WW-domain interaction. EBOV VP40 peptide interactions with WW1 of Rsp5 (square A, green boxes), WW3 of Nedd4 (square B, purple boxes), WW1 of ITCH (square C, yellow boxes), and the BAG3 WW domain (square G, red oval) are highlighted.

The “proline-rich” reading array represents a powerful tool that we have used previously to identify WW-domain proteins that physically and functionally bind to viral PPxY motifs (Fig 1) [37,39]. Here, we used biotinylated peptides containing either the eVP40 WT PPxY motif (MRRVILPTA**PPEY**MEAI) or an eVP40 PPxY mutant motif (MRRVILPTA**AAEA**MEAI) to screen 115 GST-WW domain and 40 GST-SH3 domain containing proteins (Fig 1). While the PPxY mutant eVP40 peptide did not interact with any of the arrayed WW or SH3 domains, the WT eVP40 peptide bound robustly to a number of host WW domains, including previously identified proteins Nedd4, Rsp5 (yeast ortholog of Nedd4), and ITCH (Fig 1B). Intriguingly, the WT eVP40 peptide also bound to a select number of WW-domains not identified previously, including the WW-domain of host protein BAG3 (Fig 1B, panel G).

**Fig 2:**
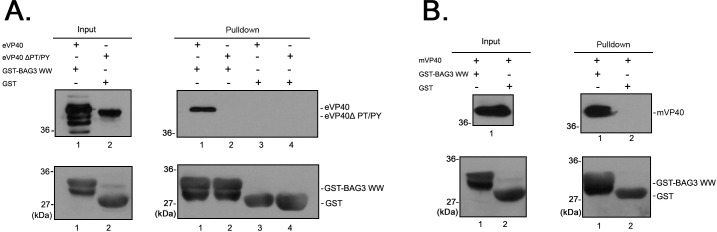
VP40/BAG3 GST pulldown assay. **A)** Extracts from HEK293T cells transfected with eVP40-WT or eVP40-ΔPT/PY plasmids were incubated with GSH beads conjugated with GST-BAG3WW or GST alone. Input and pulled-down proteins were detected by Western blotting using anti-eVP40 or anti-GST antisera. **B)** Extracts from HEK293T cells expressing flag-tagged mVP40-WT were incubated with GSH beads conjugated with GST-BAG3WW or GST alone. Input and pulled-down proteins were detected by Western blotting using anti-flag or anti-GST antisera.

We used purified GST-WW domain fusion proteins and a GST pull down assay to confirm the specificity of the BAG3-VP40 interaction (Fig 2). Briefly, protein extracts from HEK293T cells expressing either eVP40-WT, eVP40-ΔPT/PY (PPxY deletion mutant), or mVP40-WT were incubated with beads containing GST alone or GST-BAG3WW (Fig 2). Input and pulldown proteins were detected by Western blot. Indeed, we found that GST-BAG3WW, but not GST alone, pulled down eVP40 in a PPxY-dependent manner (Fig 2A, top right panel), and also pulled down mVP40 that contains a single PPxY L-domain motif (Fig 2B, top right panel). In sum, these data indicate that, compared to other PPxY-WW domain interactions interrogated previously [21], the eVP40 PPxY motif possesses a relatively high and significant degree of specificity, suggesting that BAG3 is likely a novel, biologically relevant interactor with VP40 of EBOV and MARV.

### Co-IP of VP40 and BAG3

**Fig 3:**
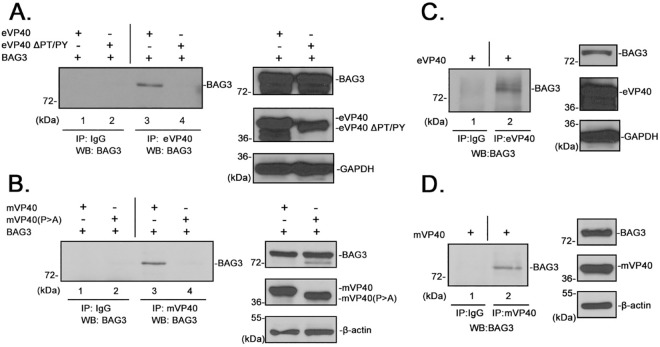
BAG3 interacts with eVP40 in a PPxY-dependent manner. **A)** Extracts from HEK293T cells transfected with eVP40 or eVP40-ΔPT/PY plus BAG3-WT were first immunoprecipitated (IP) with either normal rabbit IgG or polyclonal anti-eVP40 antisera as indicated. BAG3 was detected in the precipitates by Western blot (WB) using mouse anti-*myc* antiserum. Expression controls for eVP40-WT, eVP40-ΔPT/PY, His-myc-tagged BAG3 and GAPDH are shown. **B)** Extracts from HEK293T cells transfected with Flag-tagged mVP40 or mVP40(P>A) plus BAG3-WT were first immunoprecipitated (IP) with either normal mouse IgG or anti-Flag antisera as indicated. BAG3 was detected in the precipitates by Western blot (WB) using rabbit anti-His antiserum. Expression controls for mVP40, mVP40(P>A), His-myc-tagged BAG3 and β-actin are shown. **C)** Extracts from HEK293T cells transfected with eVP40-WT alone were first immunoprecipitated with either normal rabbit IgG or polyclonal anti-eVP40 antisera as indicated. Endogenous BAG3 was detected in the precipitates by Western blot using polyclonal anti-BAG3 antiserum. Expression controls for eVP40-WT, endogenous BAG3, and GAPDH are shown. **D)** Extracts from HEK293T cells transfected with mVP40-WT alone were first immunoprecipitated with either normal rabbit IgG or anti-mVP40 antisera as indicated. Endogenous BAG3 was detected in the precipitates by Western blot using polyclonal anti-BAG3 antiserum. Expression controls for mVP40-WT, endogenous BAG3, and β-actin are shown.

Next, we used a co-IP approach to determine whether eVP40 and mVP40 interact with full-length BAG3 expressed in mammalian cells. To test this, HEK293T cells were co-transfected with BAG3 plus WT or PPxY mutants of eVP40 (eVP40-ΔPT/PY) and mVP40 (mVP40-P>A) (Fig 3A and 3B). Cell extracts were immunoprecipitated with non-specific IgG, anti-eVP40 antiserum, or anti-mVP40 antiserum as indicated (Fig 3A and 3B), and His-*cmyc*-tagged BAG3 was detected in precipitated samples by Western blot using either anti-*cmyc* or anti-His antisera (Fig 3A and 3B). Exogenously expressed BAG3 was detected in eVP40-WT precipitates (Fig 3A, lane 3), but was not detected in preimmune IgG, nor eVP40-ΔPT/PY precipitates (Fig 3A, lanes 1 and 4, respectively). Similarly, exogenously expressed BAG3 was detected in mVP40-WT precipitates (Fig 3B, lane 3), but was not detected in preimmune IgG, nor mVP40-P>A precipitates (Fig 3B, lanes 1 and 4, respectively). These results indicated that both eVP40 and mVP40 interact with full length BAG3 in a PPxY-dependent manner in transiently transfected HEK293T cells.

Next, we asked whether eVP40 and mVP40 could interact with full length endogenous BAG3 in HEK293T cells. HEK293T cells were transfected with either eVP40-WT (Fig 3C) or mVP40-WT (Fig 3D), and once again cell extracts were immunoprecipitated with either non-specific IgG, or anti-VP40 antiserum as indicated. Endogenous BAG3 was detected in precipitated samples by Western blot using anti-BAG3 antiserum (Fig 3C and 3D). Endogenously expressed BAG3 was detected in both eVP40-WT (Fig 3C, lane 2) and mVP40-WT (Fig 3D, lane 2) precipitates, but not in preimmune IgG precipitates (Fig 3C and 3D, lanes 1). Together, these results correlate well with those from the GST pulldown assays and indicate that eVP40 and mVP40 interact with full length BAG3 in a PPxY-dependent manner.

**Fig 4:**
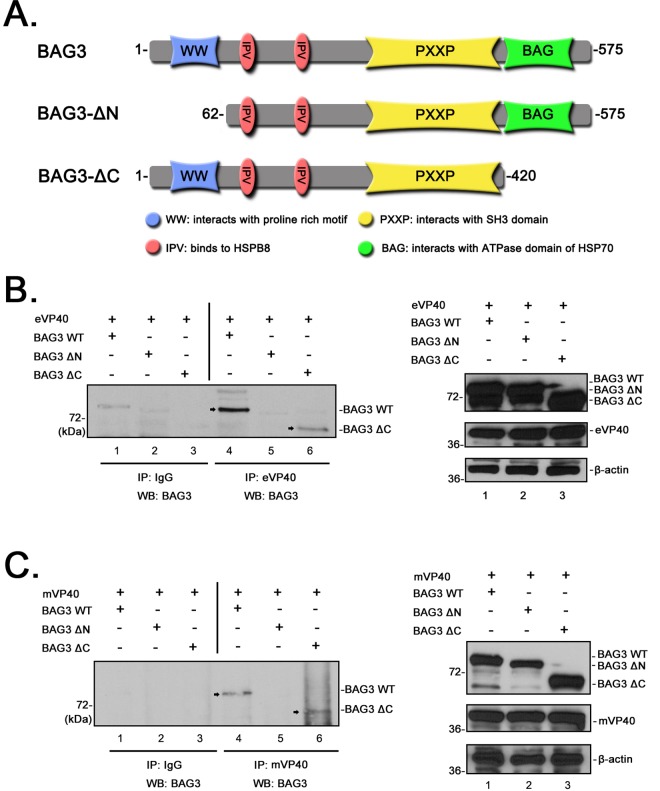
BAG3 interacts with eVP40 and mVP40 in a WW-domain dependent manner. **A)** Schematic diagram of BAG3-WT and mutants BAG3-ΔN and BAG3-ΔC, highlighting the locations of the functional domains including the single N-terminal WW-domain (blue), two IPV domains (orange), the PxxP region (yellow), and the BAG domain (green). All three proteins contain both His and *cmyc* epitope tags. **B)** Extracts from HEK293T cells transfected with the indicated plasmid combinations were first immunoprecipitated (IP) with either rabbit IgG or polyclonal anti-eVP40 antisera as indicated. BAG3-WT or mutant proteins were detected in the precipitates by Western blot (WB) using mouse anti-c*myc* antiserum. BAG3-WT (lane 4) and BAG3-ΔC (lane 6) are indicated by an arrow. Expression controls for the indicated proteins are shown. **C)** Extracts from HEK293T cells transfected with the indicated plasmid combinations were first immunoprecipitated with either mouse IgG or anti-flag (mVP40) antisera as indicated. BAG3-WT or mutant proteins were detected in the precipitates by Western blot using polyclonal anti-His antiserum. BAG3-WT (lane 4) and BAG3-ΔC (lane 6) are indicated by an arrow. Expression controls for the indicated proteins are shown.

Finally, we sought to identify the functional domains of BAG3 that mediate its interaction with eVP40 and mVP40. BAG3 contains a number of functional domains including: a single N-terminal WW-domain, two IPV regions that bind to HspB8 and function in protein quality control, multiple PxxP motifs that binds to SH3 domains, and a C-terminal BAG domain that interacts with the ATPase domain of HSP70 (Fig 4A) [41–45]. Briefly, HEK293T cells were co-transfected with eVP40 plus His/myc-tagged BAG3 WT, BAG3-ΔN (WW-domain deletion mutant), or BAG3-ΔC (BAG domain deletion mutant) (Fig 4A and 4B). Cell extracts were immunoprecipitated with either non-specific IgG, or anti-eVP40 antiserum as indicated (Fig 4B), and *cmyc*-tagged BAG3 was detected in precipitated samples by Western blot using anti-*cmyc* antiserum (Fig 4B). BAG3-WT (lane 4) and BAG3-ΔC (lane 6) were detected in eVP40 precipitates; however, BAG3-ΔN (lane 5) was not. No appreciable levels of BAG3 WT, BAG3-ΔN, and BAG3-ΔC were detected in preimmune IgG precipitates (Fig 4B, lanes 1–3). Similar results were obtained in HEK293T cells transfected with mVP40 plus the indicated BAG3 plasmids (Fig 4C). Indeed, BAG3-WT (Fig 4C, lane 4) and BAG3-ΔC (Fig 4C, lane 6) were detected in mVP40 precipitates, whereas BAG3-ΔN lacking the WW-domain was not detected (Fig 4C, lane 5). Western blots of the three BAG3 proteins, eVP40, mVP40, and actin are shown as expression controls (Fig 4B and 4C). These data demonstrate that the BAG3 WW-domain specifically mediates interactions with both eVP40 and mVP40 in mammalian cells.

### BAG3 inhibits VP40 VLP egress

**Fig 5:**
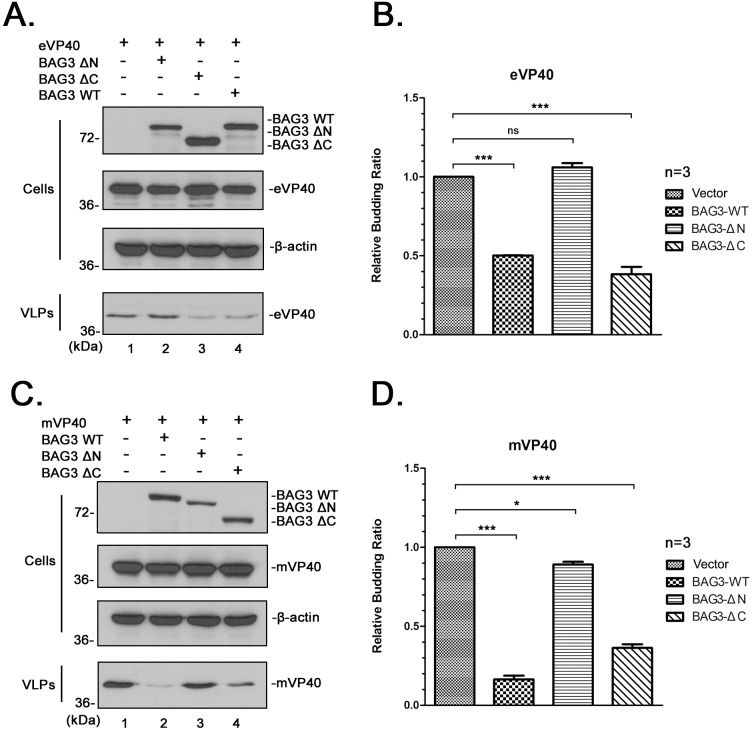
BAG3 inhibits budding of eVP40 and mVP40 VLPs in a WW-domain dependent manner. **A)** HEK293T cells were transfected with the indicated plasmid combinations, and proteins were detected in cell extracts and VLPs by Western blotting. **B)** Quantification of the relative budding ratios of eVP40 VLPs from three independent experiments. eVP40 VLPs from control cells was set to 1.0. Statistical significance was analyzed by a one-way ANOVA. ns: not significant, *** = p<0.001. **C)** HEK293T cells were transfected with the indicated plasmid combinations, and proteins were detected in cell extracts and VLPs by Western blotting. **D)** Quantification of the relative budding ratios of mVP40 VLPs from three independent experiments. mVP40 VLPs from control cells was set to 1.0. Statistical significance was analyzed by a one-way ANOVA. * = p<0.05, *** = p<0.001.

Given the highly specific and select PPxY/WW-domain interaction between VP40 and BAG3, we hypothesized that this physical interaction would have a biological consequence. To test this, we used our well-established VP40 VLP budding assay to determine whether expression of BAG3-WT, BAG3-ΔN or BAG3-ΔC would affect VP40 VLP egress. Briefly, HEK 293T cells were transfected with eVP40 or mVP40 alone, or in combination with either BAG3-WT, BAG3-ΔN or BAG3-ΔC, and both cell extracts and supernatants containing VLPs were harvested at 24 hours post transfection (Fig 5). All proteins were detected at equivalent levels in cell extracts (Fig 5A and 5C Cells). Interestingly, we found that expression of BAG3WT or BAG3-ΔC consistently resulted in a significant decrease in egress of both eVP40 (Fig 5A and 5B) and mVP40 (Fig 5C and 5D) VLPs. Importantly, we did not observe a significant decrease in egress of either eVP40 or mVP40 VLPs in the presence of WW-domain deletion mutant BAG3-ΔN (Fig 5A, compare lanes 1 and 2; Fig 5C, compare lanes 1 and 3). Intriguingly, the inhibitory effect of BAG3 appeared to be more pronounced on budding of mVP40 VLPs (Fig 5D), compared to that on eVP40 VLPs. This may reflect the presence of a single PPxY L-domain within mVP40 in contrast to the presence of overlapping PTAP and PPxY motifs within eVP40.

**Fig 6:**
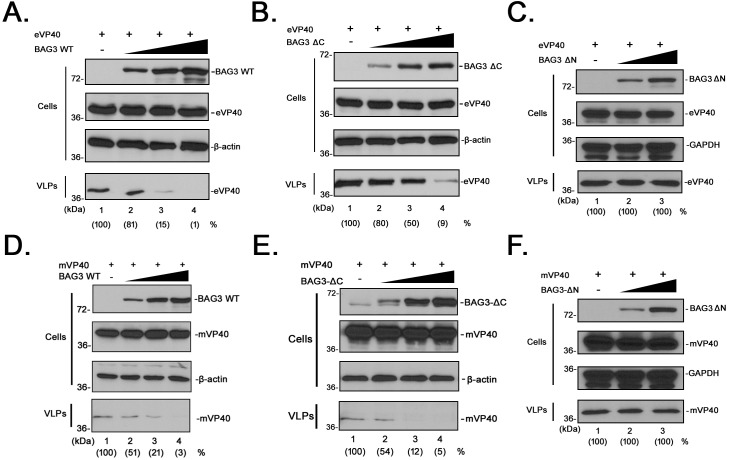
BAG3 inhibits eVP40 and mVP40 VLP egress in a dose-dependent manner. HEK293T cells were transfected with a constant amount of eVP40 plus vector (-), or increasing amounts of BAG3-WT **(A)**, BAG3-ΔC **(B)**, or BAG3-ΔN **(C)**. The indicated proteins were detected in cell extracts and VLPs by Western blotting. eVP40 VLP production from control cells (lane 1) was set at 100%, and the numbers in () represent relative VLP budding compared to the control. HEK293T cells were transfected with a constant amount of mVP40 plus vector (-), or increasing amounts of BAG3-WT **(D)**, BAG3-ΔC **(E)**, or BAG3-ΔN **(F)**. The indicated proteins were detected in cell extracts and VLPs by Western blotting. mVP40 VLP production from control cells (lane 1) was set at 100%, and the numbers in () represent relative VLP budding compared to the control.

To determine whether the novel inhibitory effects of BAG3 on VP40 VLP egress were dose-dependent, we transfected HEK293T cells with a constant amount of eVP40 or mVP40 plus increasing amounts of BAG3-WT, BAG3-ΔN or BAG3-ΔC. Cell extracts and supernatants were harvested as described above (Fig 6). Appropriate expression levels for all proteins were confirmed by Western blotting of cell extracts (Fig 6, Cells). We observed a clear dose-dependent inhibition of both eVP40 (Fig 6A and 6B) and mVP40 (Fig 6D and 6E) VLPs in the presence of increasing amounts of either BAG3-WT or BAG3-ΔC. In contrast, increasing expression of WW-domain deletion mutant BAG3-ΔN had no effect on budding of either eVP40 or mVP40 VLPs (Fig 6C and 6F). Once again, the level of inhibition mediated by BAG3-WT and BAG3-ΔC appeared to be more pronounced on mVP40 VLPs compared to that on eVP40 VLPs, with mVP40 VLPs being virtually undetectable in samples expressing the highest amounts of BAG3. Taken together, these results demonstrate a functional role for BAG3 as a negative regulator of eVP40 and mVP40 VLP budding via a WW-domain dependent mechanism. To our knowledge, this is the first host WW-domain containing protein shown to inhibit filovirus VP40 egress.

### Suppression of endogenous BAG3 enhances VP40 VLP egress

**Fig 7:**
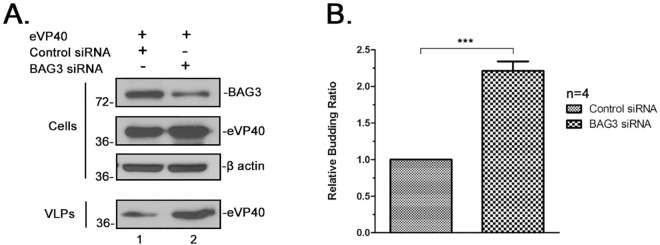
siRNA knockdown of BAG3 enhances eVP40 VLP egress. **A)** HEK293T cells were transfected with eVP40 plus either random (control) or BAG3-specific siRNA as indicated. Proteins were detected in cell extracts and VLPs by Western blotting. eVP40 VLPs from control cells (lane 1) was set at 1.0. **B)** Quantification of the relative budding ratio of eVP40 VLPs from four independent experiments. Statistical significance was analyzed by a student t test, *** = p<0.001.

We next asked whether knockdown of endogenous BAG3 would result in an increase in VP40 VLP egress. For this, we used an siRNA approach to knockdown levels of endogenous BAG3 in HEK293T cells expressing eVP40 (Fig 7). HEK293T cells were transfected with eVP40 plus either random or BAG3-specific siRNAs, and both cell extracts and VLPs were harvested and analyzed by Western blotting (Fig 7). We were able to achieve suppression of endogenous BAG3 by approximately 60% (Fig 7A, Cells), which led to a reproducible and significant increase in eVP40 VLP egress compared to control siRNA samples (Fig 7A and 7B). It should be noted that a similar ~3-fold increase was also observed for mVP40 VLPs from cells treated with BAG3-specific siRNAs. These results correlate well with those described above (Figs 5 and 6) and further confirm the inhibitory effect of BAG3 on VP40-mediated budding.

### BAG3 expression alters the intracellular localization of eVP40

**Fig 8:**
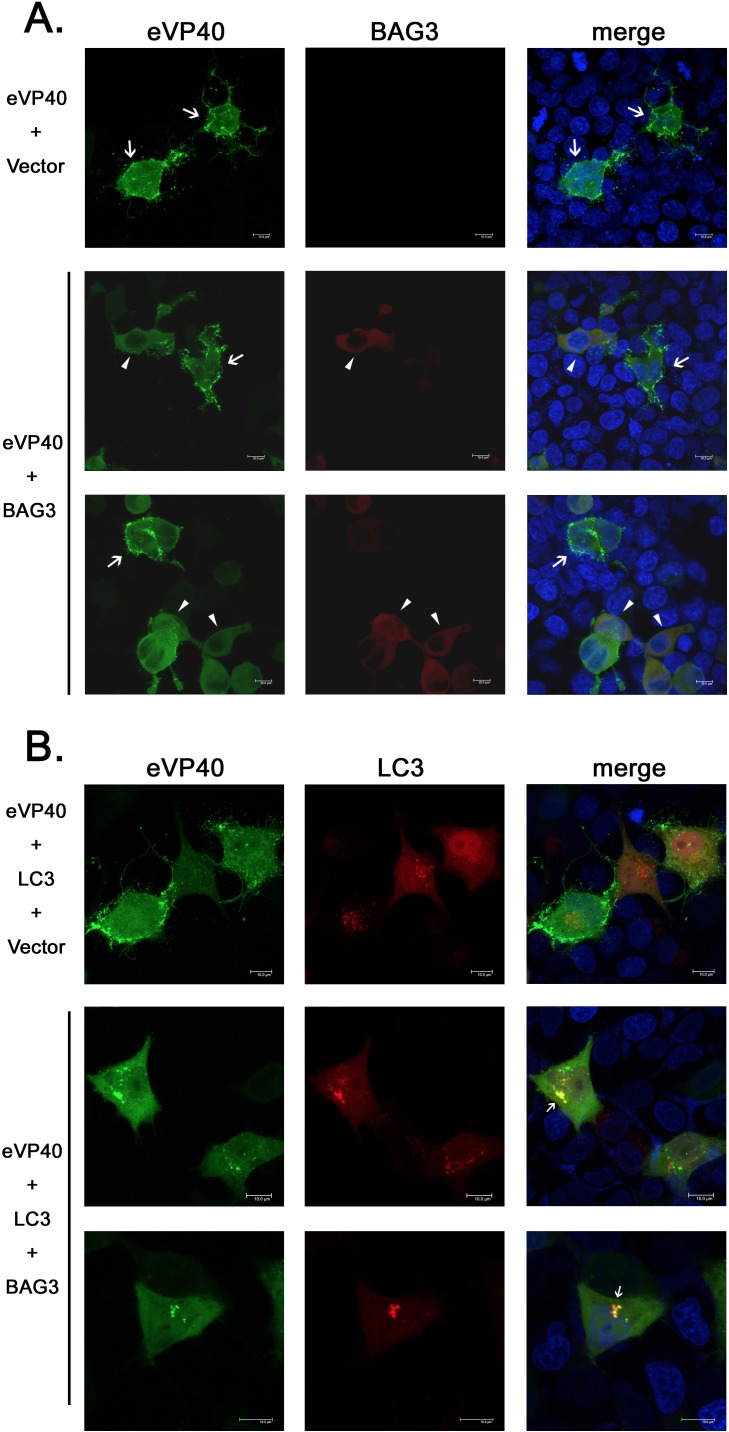
BAG3 alters the intracellular localization of eVP40 in live cells. **A)** HEK293T cells were transfected with GFP-eVP40 (green) plus either vector, or BAG3-mCherry (red), and cells were imaged at 24 hours post transfection using a Leica SP5 FLIM inverted confocal microscope. Representative images are shown with arrows highlighting the typical localization pattern of GFP-eVP40 at the plasma membrane and in PM projections, while arrowheads highlight the altered diffuse cytoplasmic localization pattern of eVP40 observed in BAG3 expressing cells. Cell nuclei were stained with NucBlue. Scale bars = 10μm. **B)** HeLa cells were transfected with GFP-eVP40 (green) plus mCherry-LC3 (red) and vector alone (top row), or BAG3 (bottom two rows), and cells were imaged at 24 hours post transfection using a Leica SP5 FLIM inverted confocal microscope. Representative images are shown with white arrows highlighting the colocalization of GFP-eVP40 and mCherry-LC3 in aggresomes. Cell nuclei were stained with NucBlue. Scale bars = 10μm.

As efficient egress of VLPs requires localization and self-assembly of VP40 at the plasma membrane (PM), we next asked whether BAG3-mediated alterations in VP40 localization correlated with egress inhibition. For this, we utilized confocal microscopy of live HEK293T cells transfected with a GFP-eVP40 fusion construct in the absence (vector alone) or presence of a BAG3-mCherry fusion construct (Fig 8A). Representative images of live cells transfected with GFP-eVP40 alone revealed the expected pattern of expression in the cytoplasm with pronounced localization around the cell periphery and in PM projections (Fig 8A, top row). In contrast, the pattern of GFP-eVP40 changed in cells co-expressing BAG3-mCherry, resulting in a more diffuse pattern of cytoplasmic localization with little to no PM projections (Fig 8A, middle and bottom rows). Indeed, this contrast in VP40 localization can be visualized in neighboring cells expressing GFP-eVP40 plus either low or high levels of BAG3-mCherry (Fig 8A, middle row). Thus, these representative live cell images suggest that localization and accumulation of eVP40 at the site of budding at the PM and in PM projections is reduced in the presence of BAG3.

As BAG3 can sequester target proteins into aggresomes during CMA, we next sought to determine whether this altered localization pattern of eVP40 was due in part to sequestration of eVP40 by BAG3 into aggresomes. To examine this possibility, we utilized confocal microscopy of live HeLa cells transfected with GFP-eVP40 + BAG3-WT + mCherry-tagged human microtubule-associated light chain-3 (LC3) protein, a well-characterized marker for aggresomes (Fig 8B) [46,47]. Representative images of live HeLa cells once again revealed the typical pattern of GFP-eVP40 predominantly at the PM in cells expressing mCherry-LC3, but lacking expression of BAG3-WT (Fig 8B, top row). Once again, the altered and more diffuse cytoplasmic pattern of GFP-eVP40 was observed in cells co-expressing BAG3-WT and mCherry-LC3; however, in addition, a fraction of GFP-eVP40 was observed to co-localize with mCherry-LC3 in puncta most likely representing cellular aggresomes (Fig 8B, bottom two rows). Together, these representative live cell confocal images suggest that the CMA function of BAG3 sequesters a fraction of eVP40 away from the PM and into aggresomes, leading to a reduction in VLP egress.

### BAG3 expression reduces PM localization of eVP40 and mVP40

**Fig 9:**
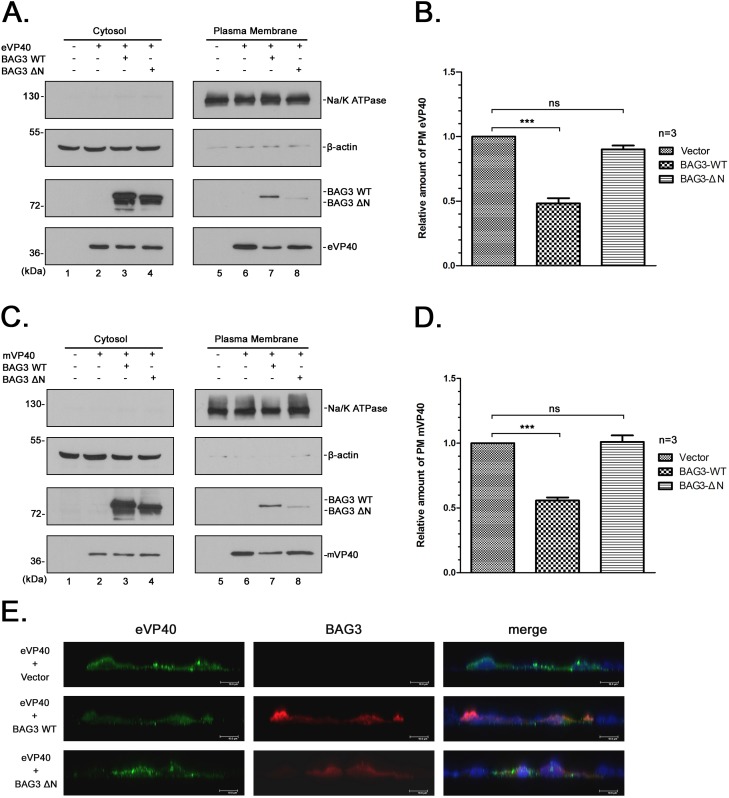
BAG3 sequesters VP40 away from the plasma membrane. HEK293T cells were mock-transfected or transfected with eVP40 **(A)** or mVP40 **(C)** plus either BAG3-WT, or BAG3-ΔN as indicated. Cytosol and plasma membrane (PM) fractions were isolated at 24 hrs post-transfection, and the indicated proteins were detected by Western blotting. β-actin served as a control protein for the cytosol fraction, whereas Na/K ATPase served as a control protein for the PM fraction. The amount of VP40 in the PM fraction in control cells (lanes 6) was set at 100% (bar graph). Quantification of the relative amount of PM-associated eVP40 **(B)** or mVP40 **(D)** from three independent experiments is shown. Statistical significance was analyzed by one-way ANOVA. ns: not significant, *** = p<0.001. **E)** HEK293T cells were transfected with eVP40 plus vector, BAG3-WT, or BAG3-ΔN as indicated. Cells were fixed at 24 hrs post-transfection, and then incubated with rabbit anti-eVP40 antiserum and mouse anti-myc antiserum (to detecting BAG3-WT and BAG3-ΔN). Cells were then stained with Alexa Fluor 488 goat anti-rabbit and 594 goat anti-mouse secondary antibodies. Microscopy was performed using a Leica SP5 FLIM inverted confocal microscope and XZY scanning. Representative images displaying eVP40 (green) and BAG3-WT (red) or BAG3-ΔN (red) localized at the PM are shown. Cell nuclei were stained with NucBlue. Scale bars = 10μm.

To further support the imaging data described above, we used a biochemical approach to determine whether the levels of eVP40 and mVP40 in PM fractions of transfected cells would be reduced in the presence of BAG3-WT, but not in the presence of BAG3-ΔN. For this, HEK293T cells were transfected with vector (pCAGGS) alone, VP40 + vector, VP40 + BAG3-WT, or VP40 + WW-domain deletion mutant BAG3-ΔN, and both cytosol and PM fractions were harvested and subjected to Western blot analysis (Fig 9). NA/K ATPase was used as a positive expression control for the PM fraction, and β-actin was used as a cytosol control (Fig 9A and 9C). As expected, both eVP40 and mVP40 were detected at equivalent levels in all cytosol fractions (Fig 9A and 9C, lanes 2–4); however, the levels of eVP40 and mVP40 in PM fractions from cells expressing BAG3-WT were consistently reduced by 2–3 fold (Fig 9A and 9C, lane 7; and Fig 9B and 9D) compared to that detected in the PM fractions of cells expressing VP40 alone (lanes 6) or VP40 + BAG3-ΔN (lanes 8).

In addition to cell fractionation studies, we examined eVP40 on the cell surface using indirect immunofluorescence and XZY scanning of confocal microscopy (Fig 9E). As expected, eVP40 alone localized robustly to the PM (Fig 9E, top row). In contrast, eVP40 localization at the PM was less pronounced in the presence of BAG3-WT compared to control cells (Fig 9E, middle row). Importantly, the PM localization pattern of eVP40 in the presence of BAG3-ΔN (Fig 9E, bottom row) was virtually identical to that in cells expressing eVP40 alone (top row). The levels of eVP40, BAG3-WT and BAG3-ΔN detected at the cell surface by indirect immunofluorescence and confocal microscopy correlate well with results from the cell fractionation studies. Taken together, our results suggest that the general mechanism by which BAG3 inhibits VLP egress is unique and involves sequestration of a fraction of eVP40 away from the PM and into aggresomes in a PPxY/WW domain-dependent manner. Although we focused on the PM, it is important to note that we cannot completely rule out the possibility that sequestration of eVP40 away from internal membranes may also contribute to the mechanism by which BAG3 inhibits VLP egress.

### BAG3 inhibits egress of infectious recombinant virus VSV-M40

**Fig 10:**
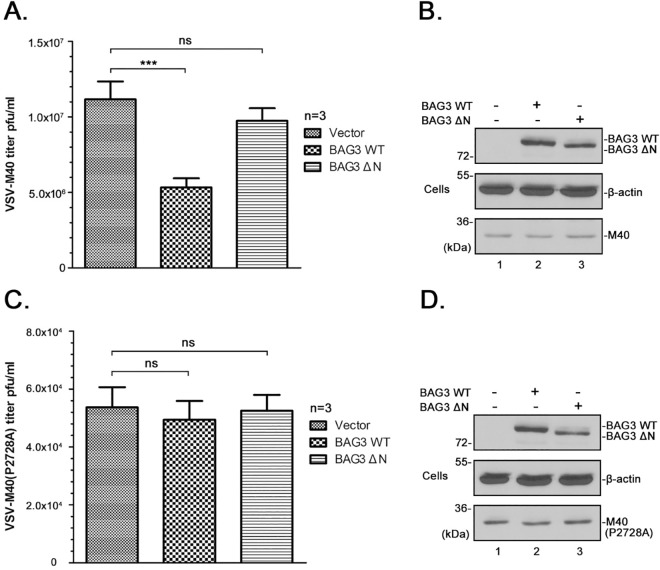
BAG3 inhibits egress of infectious recombinant virus VSV-M40. HEK293T cells were first transfected with vector alone, BAG3-WT or BAG3-ΔN for 24 hours, and then infected with recombinant virus VSV-M40 **(A)** or VSV-M40-P2728A **(C)** at a MOI of 0.1 for 8 hours. Supernatants were harvested and virus titers were determined by standard plaque assay on BHK-21 cells. Each bar represents the average of three independent experiments performed in duplicate. Statistical significance was analyzed by one-way ANOVA. ns: not significant, *** = p<0.001. The indicated proteins from VSV-M40 **(B)** or VSV-M40(P2728A) **(D)** infected cell extracts were detected by Western blotting.

Finally, we sought to determine whether expression of BAG3 would inhibit egress of infectious virus. Toward this end, we utilized our live infectious VSV recombinants; VSV-M40 and VSV-M40-P2728A [10]. Recombinant VSV-M40 expresses the WT L-domain motifs (PTAPPEY) and flanking residues from eVP40 in place of the L-domain of VSV M protein and buds efficiently, whereas recombinant VSV-M40-P2728A expresses mutated eVP40 L-domain motifs (PTAAAEY) and is budding defective [10]. Briefly, HEK293T cells were first transfected with vector alone, BAG3-WT, or BAG3-ΔN for 24 hours, and then infected with either VSV-M40 or VSV-M40-P2728A at an MOI of 0.1 for 8 hours (peak time of budding). Infected cell extracts were analyzed by Western blot for expression controls, and virus production was quantified by standard plaque assay (Fig 10). We found that the levels of infectious VSV-M40 released from mock- and BAG3-ΔN-transfected cells were virtually identical; however, titers of VSV-M40 released from cells expressing BAG3-WT were consistently and significantly reduced by >50% compared to controls (Fig 10A). In contrast, expression of BAG3-WT did not have any significant effect on budding of mutant VSV-M40-P2728A (Fig 10C). Importantly, these data demonstrate that the inhibitory effect of BAG3 on budding extends to infectious virus, and confirms the involvement of the viral PPxY/BAG3 WW-domain interaction in this negative regulatory mechanism.

## Discussion

As the major filovirus matrix protein, VP40 plays a central role in directing virion assembly and egress from infected cells. Three minimal functional domains of VP40 are required for efficient VLP egress, including a membrane (M) binding region, a self-interaction (I) domain, and one or more late (L) domain motifs. The L-domain motifs hijack or recruit specific host cell proteins that facilitate or promote efficient virus-cell separation [3,4,6,48–53]. Here, we have identified host WW-domain containing protein BAG3 as a novel interactor with the PPxY L-domain motif of both eVP40 and mVP40. Moreover, we confirmed the physical and functional interaction between the WW-domain of BAG3 and the viral PPxY L-domain motif by using L-domain and WW-domain mutants in GST-pulldowns, co-immunoprecipitation, siRNA analysis, and/or VLP/virus budding assays. Intriguingly, unlike previously identified host WW-domain proteins that interact with VP40, including Nedd4 [2,28,32,35] and ITCH [37], BAG3 is the first host WW-domain interactor to negatively regulate egress of eVP40 and mVP40 VLPs, as well as infectious virus containing the eVP40 PPxY L-domain motif.

BAG3 is a member of the BAG family of proteins (BAG1-BAG6) which are characterized by a BAG domain that interacts with the ATPase domain of heat shock protein (HSP) 70 [54]. BAG3 is the only member of this family that contains a single N-terminal WW-domain. As a co-chaperone and cell survival protein, BAG3 regulates multiple cell pathways, including, apoptosis, autophagy, cell development and cytoskeleton organization [45,55,56]. Indeed, BAG3 is induced under conditions of cell stress and plays a major role in sequestering misfolded and/or foreign proteins to the proteasome for degradation by CMA [45,55,56]. Our data imply that the CMA function of BAG3 acts as a novel host defense/response mechanism to sequester a fraction of VP40 from the site of budding at the PM and into aggresomes, thus reducing VLP/virus egress and spread. Whether sequestration of VP40 away from the PM results in degradation of VP40 remains to be determined. Interestingly, selective autophagy of the endoplasmic reticulum (ER-phagy) was recently shown to regulate EBOV replication in murine cells [57].

BAG3 has been associated with the lifecycles of other RNA and DNA viruses including, HIV-1, varicella zoster virus, herpes simplex virus, African swine fever virus, papillomavirus, polyomaviruses, coronavirus, adenovirus, and Epstein Barr virus [58–69]. However, in contrast to our findings with VP40, BAG3 primarily exerts positive effects on the lifecycles of these other viruses. For example, Gout et. al [63] reported that the adenovirus penton base protein interacted with the WW-domain of BAG3 via its PPxY motif to promote adenovirus entry and virus progeny production.

Results from cell fractionation and confocal microscopy suggest that the mechanism by which BAG3 inhibits VP40-mediated egress involves, at least in part, relocalization of VP40 away from the site of budding at the PM and accumulation of a portion of VP40 into LC3-containing aggresomes, which are visualized more definitively in HeLa cells rather than HEK293T cells. Indeed, BAG3 has been shown to regulate an aggresome-targeting pathway by interacting with the microtubule-motor dynein to selectively direct target proteins to the aggresome [56,59,70]. A more precise determination of whether sequestration of VP40 correlates with its degradation, and/or whether BAG3 may disrupt trafficking of VP40 along the cytoskeletal architecture of the cell to the PM, remains to be determined. Since BAG3 interacts with the viral PPxY motif, we cannot completely rule out the possibility that BAG3 may competitively inhibit VP40 from interacting with other host WW-domain containing proteins such as E3 ubiquitin ligases, Nedd4 and ITCH, which may impair ubiquitination and subsequent egress of VP40 VLPs. This is likely not the primary mechanism of budding inhibition since the intracellular localization of VP40 was altered significantly in the presence of BAG3 as judged by PM fractionation and confocal microscopy.

In sum, we identified BAG3 as a novel, WW-domain interactor with the PPxY motif of VP40 leading to inhibition of VLP and infectious virus egress in a PPxY/WW-domain dependent manner. As a stress-induced, cell survival protein, BAG3 may represent a key component of a novel host defense mechanism to dampen virus egress via CMA and protein sequestration. These findings provide new insights into the roles that host proteins play in regulating filovirus VP40-mediated egress, and a more comprehensive understanding of these virus-host interactions may be helpful in the design of future antiviral therapies. For example, it may be possible to identify small molecules that could bind to VP40 in a manner that mimics the inhibitory effect of BAG3. Alternatively, the WW domain of BAG3 alone (per se) could be used to inhibit VLP and virus egress as documented previously for *cis*-expressed YAP WW domain that inhibited PPxY-mediated budding of Rous Sarcoma virus [71].

## Materials and Methods

### Cell lines and plasmids

HEK293T (American Type Culture Collection; ATCC), HeLa (American Type Culture Collection; ATCC), and BHK-21 (American Type Culture Collection; ATCC) cells were maintained in Dulbecco’s modified Eagle’s medium (DMEM) (CORNING) supplemented with 10% fetal bovine serum (FBS) (GIBCO), penicillin (100U/ml)/streptomycin (100μg/ml) (INVITROGEN) and the cells were grown at 37°C in a humidified 5% CO_2_ incubator. The plasmids encoding eVP40-WT, eVP40-ΔPT/PY were described previously [2,72,73]. Flag-tagged mVP40-WT and PPxY mutant mVP40(P>A) were kindly provided by S. Becker (Institut für Virologie, Marburg, Germany). The pcDNA6 myc-His-BAG3-WT (1–575), pcDNA6 myc-His-BAG3-ΔN (62–575) and pcDNA6 myc-His-BAG3-ΔC (1–420) plasmids were kindly provided by K. Khalili (Temple University). Plasmid pDEST-mCherry-BAG3 was kindly provided by E. Sjøttem (University of Tromsø). mCherry-hLC3B-pcDNA3.1 was a gift from David Rubinsztein (Addgene plasmid # 40827) [74].

### PPxY-WW domain reading array screen

The proline rich motif “reading” array consisted of almost all known WW domains (115 domains) from mammalian proteins (and yeast). We prepared biotinylated peptides harboring either the EBOV VP40 WT PPxY motif (MRRVILPTA**PPEY**MEAI) or mutated PPxY motif (MRRVILPTA**AAEA**MEAI). Both of the biotinylated peptides were fluorescently labeled and used to screen the specially prepared “proline-rich” reading array.

### GST-pulldown assay

GST alone and GST-BAG3 WW domain fusion protein were expressed in BL-21 cells and subsequently conjugated to glutathione (GSH) beads (GE HEALTHCARE). HEK293T cells were transfected with eVP40-WT, eVP40-ΔPT/PY or flag-tagged-mVP40, respectively. At 24 hours after transfection, the cell extracts were incubated with the GSH beads described above at 4°C for 4 hours with continuous rotating. The proteins complexes were pulled down with beads via centrifugation. The rabbit eVP40 antiserum (PROSCI), mouse anti-flag monoclonal antibody (SIGMA), mouse anti-GST monoclonal antibody (SIGMA) were used to detect eVP40-WT, eVP40-ΔPT/PY, mVP40, GST, or GST-BAG3 WW proteins in input and pulldown samples by Western blotting.

### Immunoprecipitation assay

HEK293T cells were transfected with the indicated plasmids combinations using Lipofectamine reagent (INVITROGEN). At 24 hours post transfection, cells were harvested and lysed, and cell extracts were incubated with either rabbit or mouse IgG, eVP40, or mVP40 specific antisera as indicated. Protein A or G agarose beads were then added to the mixtures and incubated overnight at 4°C. After incubation, beads were collected via centrifugation and washed 4X. Proteins were then detected by Western blotting with polyclonal anti-BAG3 (Proteintech), polyclonal anti-His (Cell Signaling), or monoclonal anti-*cmyc* (Millipore) antisera as indicated.

### VLP budding assay and BAG3 titration

Filovirus VP40 VLP budding assays in HEK293T cells were described previously [2,13,35,72,73]. eVP40 and mVP40 proteins in VLPs and cell extracts were detected by SDS-PAGE and Western blotting, and quantified using NIH Image-J software. The anti-eVP40 antiserum was used to detect eVP40-WT and eVP40-ΔPT/PY mutant, and anti-flag monoclonal antibody was used to detect flag-tagged mVP40. For BAG3 titration experiments, HEK293T cells were transfected with 0.1μg of eVP40 or mVP40 and increasing amounts of BAG3-WT or BAG3-ΔC (0.1, 0.5, 1.0μg), or BAG3-ΔN (0.1 and 1.0μg). The total amounts of transfected DNA were equivalent in all samples. Supernatants and cell extracts were harvested at 24 hours post transfection.

### siRNA knockdown

HEK293T cells seeded in 6 well plates were transfected with human BAG3-specific or random siRNA (DHARMACON) at a final concentration of 100nM per well using Lipofectamine reagent (INVITROGEN). At 24 hours post transfection, cells were transfected again with 0.2μg of eVP40 or mVP40 plasmid. VLPs and cell extracts were harvested at 24 hours post transfection, and proteins were detected by Western blotting.

### Live cell imaging

HEK293T cells were transfected with GFP-eVP40 plus mCherry-BAG3 or vector (pCAGGS), and cells were monitored by Leica SP5 FLIM inverted confocal microscope at 24 hrs post-transfection. HeLa cells were transfected with GFP-eVP40 plus mCherry-LC3 and either BAG3-WT or vector alone, and cells were monitored by Leica SP5 FLIM inverted confocal microscope at 24 hrs post-transfection. Cell nuclei were stained by NucBlue live cell ready probes (LIFE THCHONOLOGIES). The intracellular localization of GFP-eVP40, mCherry-BAG3, and mCherry-LC3 in the live cells were imaged using XYZ scanning. To generate series images through the whole cell, serial optical planes of focus (at approximately 1μm intervals) were taken through the Z stacks from the top to bottom of the cell, and the collected images were merged into one using the Leica microsystems (LAS AF) software.

### Indirect Immunofluorescence assay

HEK293T cells were transfected with the indicated plasmid combinations. At 24 hours post transfection, cells were washed with cold PBS and fixed with 4% formaldehyde for 15 min at room temperature, then permeabilized with 0.2% Triton X-100. After washing 3X with cold PBS, cells were incubated with polyclonal anti-eVP40 antiserum and mouse anti-*cmyc* antiserum to detect his-myc tagged BAG3 and its mutants. Next, cells were stained with Alexa Fluor 488 goat anti-rabbit and 594 goat anti-mouse secondary antibodies (LIFE TECHONOLOGIES). Cell nuclei were stained with NucBlue fixed cell ready probes (LIFE TECHONOLOGIES). Microscopy was performed using a Leica SP5 FLIM inverted confocal microscope and an XZY scanning model. Serial optical planes of focus were taken on the Y-axis and the collected images were merged into one by using the Leica microsystems (LAS AF) software.

### Cytosol and plasma membrane protein fractionation

HEK293T cells were transfected with indicated plasmid combinations, and cells were scraped and washed with cold PBS at 24 hours post-transfection. Cells were then collected via low speed centrifugation. The cytosol, organelle membrane and plasma membrane protein fractions were isolated sequentially using the “Minute plasma membrane protein isolation kit” (INVENT) following the manufacturer's instructions. Proteins within the cytosol and plasma membrane fractions were detected via SDS-PAGE and Western blotting. The β-actin and sodium potassium ATPase were used as cytosol and plasma membrane controls and were detected using mouse anti β-actin (SIGMA) and rabbit anti Na/K ATPase (ABCAM) monoclonal antibodies.

### Transfection/Infection assays

HEK293T cells were first transfected with BAG3, BAG3-ΔN or vector for 24 hours, and then subsequently infected with either VSV-M40 or VSV-M40-P2728A at a MOI of 0.1. Supernatants and infected cell extracts were harvested at 8 hours post-infection. Released VSV-M40 and VSV-M40-P2728A virions were titrated in duplicate via standard plaque assay on BHK-21 cells. Cellular proteins were detected by Western blotting using specific antibodies.
