# Correction: Chaperone-Mediated Autophagy Protein BAG3 Negatively Regulates Ebola and Marburg VP40-Mediated Egress

**DOI:** 10.1371/journal.ppat.1006519

**Published:** 2017-07-19

**Authors:** Jingjing Liang, Cari A. Sagum, Mark T. Bedford, Sachdev S. Sidhu, Marius Sudol, Ziying Han, Ronald N. Harty

The authors would like to correct the use of the term “Chaperone-Mediated Autophagy (CMA)” throughout the article. Since the authors did not directly analyze autophagy processes in this article, all mentions of the specific process of “Chaperone-Mediated Autophagy (CMA)” should be removed from the article.

The term “Chaperone-Mediated Autophagy” appears ten times throughout the article: once in the title, twice in the Abstract, once in the Author Summary, once in the Introduction, twice in the Results, and three times in the Discussion.

The title should read “Co-Chaperone Protein BAG3 Negatively Regulates Ebola and Marburg VP40-Mediated Egress.”

Throughout the text, the use of “Chaperone-Mediated Autophagy (CMA)" should be deleted or replaced with "co-chaperone" depending on the context of the sentence.

All changes to the original article are detailed in a marked-up PDF included as S1 File. A clean Word document version of the revised article is included as S2 File.

The authors confirm that these changes do not alter their findings.

## Supporting information

S1 File Marked-up PDF detailing changes. (PDF)

S2 File Clean Word version of revised article. (DOCX)

## References

[1] Liang J, Sagum CA, Bedford MT, Sidhu SS, Sudol M, Han Z, et al (2017) Chaperone-Mediated Autophagy Protein BAG3 Negatively Regulates Ebola and Marburg VP40-Mediated Egress. PLoS Pathog 13(1): e1006132 https://doi.org/10.1371/journal.ppat.1006132 28076420 10.1371/journal.ppat.1006132 PMC5226679

